# Endoscopic Endonasal Skull Base Surgery Complication Avoidance: A Contemporary Review

**DOI:** 10.3390/brainsci12121685

**Published:** 2022-12-08

**Authors:** Jose L. Porras, Nicholas R. Rowan, Debraj Mukherjee

**Affiliations:** 1 Department of Neurosurgery, The Johns Hopkins University School of Medicine, 600 N Wolfe St., Phipps 124, Baltimore, MD 21287, USA; 2 Department of Otolaryngology-Head and Neck Surgery, The Johns Hopkins University School of Medicine, 601 N Caroline St., Suite 6164, Baltimore, MD 21287, USA

**Keywords:** endoscopic endonasal approach, complications, quality-of-life

## Abstract

The endoscopic endonasal approach (EEA) provides a direct trajectory to ventral skull base lesions, avoidance of brain retraction, and clear visualization of cranial nerves as they exit skull base foramina. Despite these benefits, the EEA is not without complications. Here, we review published literature highlighting complications associated with the EEA including cerebrospinal fluid (CSF) leak, cranial nerve (CN) dysfunction, pituitary gland dysfunction, internal carotid artery (ICA) injury, infection, and others; we place special emphasis on discussing the prevention of these complications. As widespread adoption of the EEA continues, it becomes critical to educate surgeons regarding potential complications and their prevention while identifying gaps in the current literature to guide future research and advances in clinical care.

## 1. Introduction

The EEA for skull base surgery has developed rapidly since it was first introduced as an adjunct to the microscope for pituitary tumor resection in 1979 [1,2,3]. The EEA provides several advantages including a direct trajectory to ventral skull base lesions, avoidance of brain retraction, and visualization of CNs. Relative to traditional open approaches, the EEA may offer better neurological outcomes and shorter hospital length of stay in some instances [4,5]. The indications for EEA have expanded over the past four decades and now routinely include the resection of skull base pathologies found in the anterior, middle, and posterior cranial fossae. Additionally, as the complexity of endoscopic skull base surgery continues to increase, so too does the potential for complications. In this review, we provide an overview of potential complications from the EEA, methods for prevention, and identify gaps in the literature.

## 2. Materials and Methods

**Figure 1:**
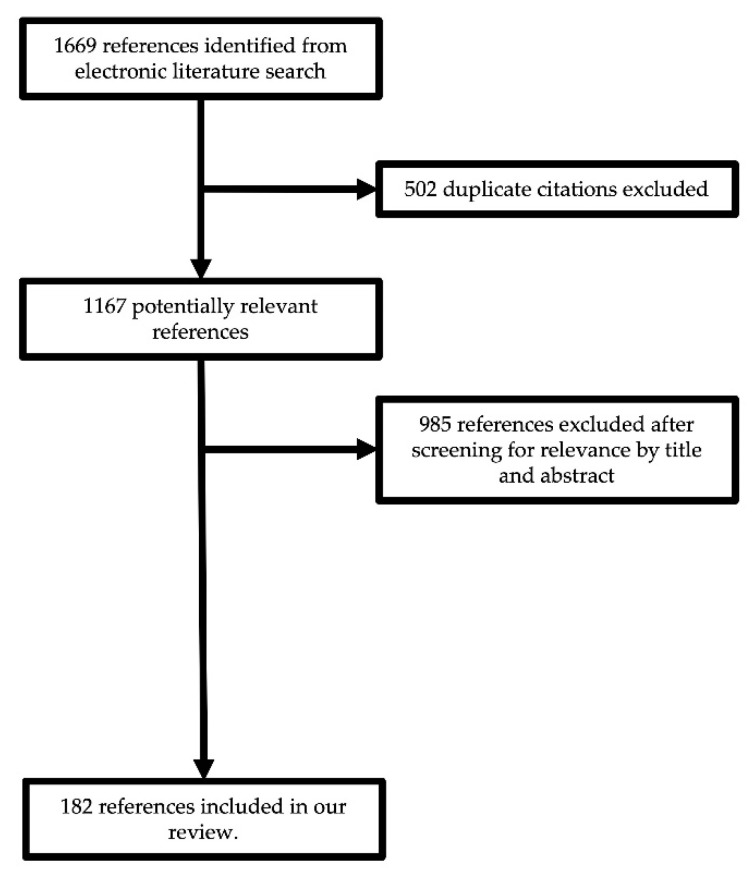
Study selection flow diagram.

To conduct a review of the complications associated with endoscopic endonasal skull base surgery, we reviewed all available literature through PubMed MEDLINE (National Library of Medicine) by searching for variations of search terms related to complications associated with endoscopic endonasal skull base surgery (e.g., CSF leak, carotid injury). We included studies from 1 January 1970 through 30 June 2022. Eligibility criteria included English language and retrospective studies, case series, case reports, or reviews. Studies describing complications unique to open or microscopic skull base surgery were not included. In total, 1669 potential studies were found after aggregating our searches. After removal of duplicates, we identified 1167 candidate studies. Remaining articles were screened for relevance by title and abstract. After manual searching of titles and abstracts, we arrived at the 182 references included in our review (Figure 1).

## 3. CSF Leak

Post-operative CSF leak is the most common post-operative complication following EEA. Early series describing the EEA were marked by post-operative CSF leak rates as high as 40% [6] though more recent series have reported leak rates as low as 2.9% [7]. A significant source of morbidity, CSF leak complications may include meningitis, symptomatic pneumocephalus, acute subdural hemorrhage, cerebellar sag, or abducens nerve palsy secondary to traction [8,9,10]. CSF leak also leads to increased hospital length of stay and risk of readmission which is especially disruptive for patients requiring adjunctive therapy for a skull base malignancy [11,12].

### 3.1. Pre-Operative Risk Factors

There are several patient risk factors associated with skull base reconstruction failure. In a retrospective review of 615 patients undergoing an EEA for an intradural lesion by Fraser et al., 35 patients had pre-operative hydrocephalus, which was found to be significantly associated with post-operative CSF leak, 71.4% of whom underwent shunt placement for CSF diversion [13]. The same authors independently assessed BMI and noted that the incidence of CSF leak was significantly higher in overweight and obese patients relative to those with a normal BMI (18.8% vs. 11.6%, *p* = 0.04) [13]. This association between elevated BMI and increased rates of post-operative CSF leak has been demonstrated in several other studies [10,14,15,16,17].

The impact of gender on risk of post-operative CSF leak is less clear. Pooled data from six institutions suggested female gender to be an independent predictor of post-operative CSF leak on multivariate analysis. However, two separate studies suggested an association between male gender and post-operative CSF leak [16,18]. Several other studies have failed to show that gender influences reconstruction outcome [10,13,19,20,21]. At this time collective data does not seem to suggest a definitive association between gender and post-operative CSF leak.

Similarly, there is not clear evidence suggesting that age is associated with post-operative CSF leak. Two multi-institutional studies demonstrated younger age was significantly associated with reconstructive failure [14,15]. Though a similar finding was reported by Dlouhy et al. [16], the opposite was found by Ivan et al. who demonstrated older patients to be at increased risk of post-operative CSF leak [10]. Several studies have found no association [13,18,19,20,21,22].

Existing literature does not clearly support an increased risk of post-operative CSF leak for patients undergoing revision pituitary surgery. In one study matching 50 patients undergoing revision pituitary surgery to those undergoing primary pituitary surgery, no difference was found in rates of post-operative CSF leak [23]. Similar findings have been demonstrated in multiple other studies [10,15,20,22]. However, further literature describing leak rates in non-pituitary revision surgery is required. 

Data is mixed regarding risk of prior radiation on post-operative CSF leak. A retrospective review of endoscopic pituitary patients from seven institutions (*n* = 1161) demonstrated a 28% post-operative leak rate compared to 5.6% in patients without prior radiation (*p* = 0.007) [14]. A separate study assessing 70 patients found an increased failure of CSF leak repair for patients having undergone prior radiation therapy (OR = 2.67, *p* = 0.047). Conversely, several other retrospective studies did not find prior irradiation to a predictor of post-operative CSF leak [10,20,22].

Surgeon experience has also been found to be a risk factor for CSF leak with most leaks occurring earlier in a surgeon’s career [22,24,25]. CSF leaks have been reported to plateau once a surgeon approaches 100 cases of experience [26]. However, the generalizability of these findings are limited as reconstructive techniques have also improved throughout the careers of many of the studied surgeons.

### 3.2. Intra-Operative Risk Factors

Risk of post-operative CSF leak varies by operative site. Sellar defects have been reported to carry an incidence of reconstructive failure ranging from 1.3% to 13.5% [7,14,16,20,23,27,28,29]. For parasellar lesions, rates of post-operative CSF leak range from 2.9% to 15.3% [13,20,24,26,30,31,32]. Many of these studies report combined sellar and parasellar lesions or pituitary lesions with suprasellar extension. Importantly, many studies also combine patients with no intraoperative CSF leak into the reported cohort, which is has an anticipated lower risk of postoperative leak as compared to cases with an intraoperative leak. Fraser et al. in contrast reported a post-operative CSF leak rate of 10% in a cohort that only included isolated sellar lesions with a documented intraoperative CSF leak requiring dural repair [13]. 

Fewer studies assessed reconstructive outcomes for anterior cranial fossa defects, with reported reconstructive failures ranging from 4% to 20.7% [9,13,18,22,27]. Posterior cranial fossa defects are considered to be the highest risk location for post-operative CSF leak given the high-flow nature of the prepontine cistern. Rates of CSF leak for clival defects are reported between 6.7% to 32.7% [9,13,22,27,33,34,35]. Limited data is available for craniocervical (odontoid) lesions, of which many are extradural (i.e., basilar invagination, rheumatoid pannus). Additionally, pedicled flaps used in other reconstructions are challenging to implement at this location. Nevertheless, reported rates of reconstruction failure for craniocervical lesions range from 0% to 5.2% [36,37,38].

Rate of flow through a CSF defect is a notable risk factor for post-operative reconstruction failure. High-flow CSF leaks are those in which a skull base defect is in direct communication with a ventricle or cistern [39]. High-flow and large CSF leaks are more likely to leak post-operatively [14,15,27,40,41]. Numerous studies support the use of a vascularized flap reconstruction over free-tissue graft when able for high-flow CSF leaks [13,14,27,42,43,44]. In studies where the patient population had mostly sellar defects (and therefore presumably lower flow defects) similar rates of CSF leak were observed between vascularized flaps or layered free grafts, suggesting that free-graft reconstructions may be similarly effective in smaller, low-flow defects [18,20,25]. Recently, Chaskes et al. have described an algorithm for sellar reconstruction after EEA, which achieved a 1.5% postoperative CSF leak rate across 582 patients [45]. The authors provide recommendations for reconstruction when there is either no leak, a low-flow leak, or a high-flow leak. They report that nearly 94% of their patient cohort were reconstructed with a single-layer repair and did not require additional packing material, a nasoseptal flap, or lumbar drainage.

The utility of routine lumbar drain (LD) placement for prophylactic CSF diversion has been a past subject of debate. Despite its advantages, LD usage is not without its drawbacks including potential meningitis, headache, pneumocephalus, epidural hematoma, presence of an additional wound, and prolonged hospitalization [46]. Many prior studies questioned the utility of lumbar drainage for reducing post-operative CSF leak, demonstrating no significant difference in post-operative leak rates [13,20,25,28,40,47,48]. However, many such studies were limited in that they considered CSF leaks from multiple etiologies, or they were retrospective in nature, thus subject to significant bias (for example, LDs being used more frequently in patients at high-risk of post-operative leak). However, more recently a prospective randomized control trial describing 170 patients (85 with prophylactic LD placement) demonstrated that patients with LD had an 8.2% rate of CSF leak compared to a 21.2% rate in the control group [49]. Critically, the rate of leak was higher for patients with larger defects and for anterior or posterior fossa defects. There was not a significant difference in post-operative leak rate for patients with suprasellar pathology who underwent LD placement (4.7% with LD, 9.5% without LD, *p* = 0.43). Whereas, for anterior fossa leaks the rates were 11.1% with LD compared to 35.3% without (*p* = 0.012) and for posterior fossa leaks the rates were 12.5% with LD compared to 30.8% without (*p* = 0.012). There was no observed effect of BMI on rates of post-operative leak. Therefore, cumulative data does not favor prophylactic use of LDs in cases with low-flow and small defects but is more favorable when large or high-flow defects are anticipated.

## 4. Optic Nerve Damage

Pituitary macroadenomas commonly present with visual field deficits (46–75%) and decreased visual acuity (14–44%) due to direct chiasmal compression [50,51,52,53]. Approximately 60% of nonfunctioning pituitary macroadenomas experience visual deficits as the main presenting symptom [54,55]. The EEA can facilitate visual improvement and earlier surgical decompression maximizes visual outcomes [56,57,58]. However, despite the ability of the EEA to improve vision, visual fields may deteriorate post-operatively for example in up to 4% of pituitary adenoma cases [59]. The visual pathways may be susceptible to direct surgical damage, vascular compromise, post-operative hemorrhage, excess packing during CSF leak repair, or mechanical injury from optic nerve prolapse into an empty sella [60]. Steps to preserve and improve visual function during surgery include minimizing nerve manipulation, identifying and relieving pressure at tether points, and preserving the vascular supply. Gross total resection is not required to achieve improved visual outcomes, likely owing to sufficient optic apparatus decompression achieved by partial resection [57,61,62]. This point underscores the role of surgeon experience in being associated with post-operative visual field improvements [63,64,65].

## 5. Other Cranial Nerve Deficits

By compressing CNs, pituitary adenomas have been associated with eye movement deficits in between 1.4% to 4.6% of cases [66,67]. Oculomotor nerve palsy is thought to arise secondary to pressure transmitted to the cavernous sinus by tumor growth or infiltration and direct compression of the nerve between the interclinoid ligament and the tumor [68,69]. Trochlear nerve palsies are generally only reported in association with other CN palsies. Total ophthalmoplegia is a rare event generally occurring in the context of pituitary apoplexy or a malignancy such as lymphoma or metastasis [70].

Among the cranial nerves, isolated abducens nerve palsies are the most common and may occur from pressure anywhere along its course secondary to lesions including pituitary adenomas, nasopharyngeal carcinomas, cholesteatomas, chordomas, chondrosarcomas, meningiomas, and others [71,72,73,74]. Minimizing the risk of abducens nerve injury requires a deep understanding of its anatomy including trajectory, location, and associated surgical landmarks [71]. For example, a petroclival lesion may grow from a lateral to medial trajectory thereby displacing the abducens toward the midline. Injury to the abducens nerve may also occur from disruptions to its blood supply such as the inferolateral trunk arising from the cavernous carotid artery [75].

Neuromonitoring during an EEA can be cost effective and positively impact patient quality of life by helping to identify CNs during surgery [76]. However, CN monitoring should be undertaken with an understanding of the benefits and limits of each modality. Free-run electromyography (fEMG) is a strategy for continuous monitoring of CNs during EEA [77,78]. In a study describing 696 extraocular CNs (III, IV, VI) and 342 lower CNs (VII, IX, X, XI, XII), fEMG activity was seen in 12% of extraocular CNs and 18% of lower CNs [79]. Patients with radiation history or undergoing a repeat surgery had increased incidence of fEMG activity likely due to adhesions. Despite this sensitivity, the absence of fEMG activity may provide a false sense of security and has limited value in predicting post-operative CN function [80,81]. Even transection of a CN may lead to only brief or no EMG activity [82]. 

Mapping techniques using triggered EMG (tEMG) resulting in compound muscle action potentials (CMAPs) are particularly useful to identify and avoid injury to CNs [83]. Changes in stimulation threshold, onset latency, and CMAP amplitude may predict post-operative CN function [81,84,85,86]. Nerve transection may be more readily identified by loss or change in previously recorded CMAP thresholds [85]. Data regarding the use of transcranial motor-evoked potentials (TMEPs) is more limited but there is correlation of corticobulbar MEP changes with post-operative CN function in large tumors that completely encompass CNs [87,88,89,90,91]. Corticobulbar MEPs may be helpful relative to tEMG during tumor bulking by providing information about CN function rather than relying on tEMG response via direct stimulation.

## 6. Pituitary Gland Dysfunction

Diabetes insipidus (DI), syndrome of inappropriate antidiuretic hormone release (SIADH), and panhypopituitarism are all potential sequelae of disruption of the hypothalamic-pituitary axis (HPA) owing to the pathology and surgical corridor associated with the EEA [92]. Macroadenomas are more commonly associated with anterior pituitary hormone axis derangements relative to microadenomas likely owing to compression of portal vessels in the infundibulum caused by tumor expansion or elevated intrasellar pressure [93,94,95]. HPA derangements that occur post-operatively may be secondary to either direct (surgical) or indirect (decompressive, vascular) manipulation [96]. While most HPA disruptions are transient, they require close clinical and laboratory monitoring to minimize risk of further complication [96,97]. Post-EEA transient DI carries a reported incidence between 4.6% to 8.7% [96,97,98,99,100].

Minimizing the risk of complications associated with HPA dysfunction starts with the pre-operative evaluation. Pre-operative low fasting morning cortisol may warrant administration of stress dose steroids at time of anesthesia induction with a subsequent taper to a maintenance dose. Patient factors including young age, greater tumor size, and tumors involving the pituitary stalk and posterior gland (e.g., Rathke’s cleft cyst and craniopharyngioma) confer a heightened risk of HPA dysfunction [97,101]. With respect to post-operative hyponatremia, a multivariate analysis suggested that only pre-operative hypopituitarism predicted post-operative hyponatremia [100].

Intraoperatively, surgical exploration of the posterior gland or traction on the infundibulum are associated with an increased risk of HPA dysregulation [102]. Pars intermedia tumors and cystic adenomas are thought to confer an elevated risk as well.

## 7. Internal Carotid Artery Injury

Injury to the internal carotid artery is a feared but uncommon complication of the EEA with an incidence between 0.016% to 1% [103,104,105,106] and a mortality rate reaching up to 10% [107]. A recent anonymous survey of skull base course attendees found that at least 20% of surveyed surgeons reported a carotid injury in their career with most occurring during tumor exposure and removal (48%) and most occurring to the parasellar carotid artery segment (39%) [108]. Management is made difficult by the restrictive corridor afforded by the EEA relative to traditional open approaches [109,110,111,112]. This may be especially true in the pediatric patient population where for example the paraclinoid intercarotid artery distance can average 0.96 cm for patients <1 years old to 1.37 cm for patients 17 years of age [113].

A critical principal in minimizing the risk of an ICA injury is recognizing when and where ICA injuries are most likely to occur. Most reported injuries to the ICA occur during the stages of skull base exposure (specifically sphenoidotomy) and tumor resection [107]. Most injuries are to the left ICA likely owing to the fact that most surgeons are right-handed [107]. While not statistically significant, rates of ICA injury have trended toward significance when divided by approach, with transsellar approaches carrying a 0.3% risk relative to a risk of 0.9% for transclival/transpterygoid approaches [103]. Injury to the C2 (petrous), C3 (paraclival), and C5 (paraclinoidal) segments have all been reported but the most commonly injured segment is C4 (parasellar) [107,108].

Review of pre-operative imaging is critical for understanding patient anatomy and for contingency planning. The bone overlying the ICA is <0.5 mm thick in 88% of specimens and may be dehiscent in 4% to 22% of anatomic specimens [114,115]. Inter-sphenoidal septations commonly attach to the ICA canal and are often readily recognized on CT [116]. Pre-operative vessel imaging can help the surgical team to understand robustness of the circle of Willis and vascular collateralization to gauge risk of ischemia should vascular injury occur. When pre-operative studies suggest circumferential involvement or adventitial invasion of the ICA, balloon test occlusion may be considered to assess vascular reserve, though this test is not without its own risk and may underestimate stroke risk [117,118].

Pathology type may also be a risk factor for ICA injury. For example, in one study of chondroid tumors, 3/142 cases (2%) experienced an intraoperative ICA injury [103]. Increased risk of ICA injury has also been reported for GH-secreting pituitary adenomas owing to more complex sphenoid sinus anatomy and a tortuous ICA [119]. Other described risk factors for ICA injury include prior surgery, prior radiation therapy, chemotherapy, and prior use of bromocriptine [103,109,120].

While detailed management of ICA injury, inclusive of hemorrhage control and repair strategies, is beyond the scope of this review, it is important to mention that part of minimizing the risk of complication from an ICA injury is preparedness. It is difficult to discern whether intraoperative adjuncts such as neuronavigation and micro-Doppler ultrasound lead to decreased rates of ICA injury given the retrospective nature and mixed reporting of ICA injury cases [105,107,114]. Regardless, some authors report no ICA injuries after the introduction of intraoperative neuronavigation and micro-Doppler ultrasound and therefore advocate for its routine use [105,121,122]. Intraoperatively, the development of sudden, high-flow arterial bleeding may suggest ICA injury [112]. Given the constraints of the EEA, a two-surgeon approach is recommended to maximize visualization [103,120,123]. ICA injury may be caused by multiple instrument types including Kerrison punches, drills, ring curettes, Blakesley, Thru-Cut forceps, microdebrider, and suction instruments [107,124].

## 8. Infection

There is a theoretical risk of infection after EEA provided the presence of a sinonasal microbiome and the potential for communication with the intracranial cavity [125]. However, in the absence of a CSF leak, the risk of meningitis or other intracranial infectious complications appears to be minimal. Failure to repair an operative CSF leak is associated with up to a 21% incidence of meningitis [44]. The risk of meningitis in EEA has been described to be directly related to presence of a post-operative leak, underscoring the importance of an adequate intraoperative CSF leak repair [9]. Provided this low risk of infection and the potential morbidity associated with antibiotic use (including allergic reaction, infectious colitis, and microbial drug-resistance), the judicious use of antibiotics for EEA is recommended. In a 2016 systematic review, the use of or duration of nasal packing was not linked to infectious complications or duration of antibiotics [126].

Most existing literature describing the role of antibiotics in EEA are retrospective and describe a wide variety of pathologies, surgical sites, and patient factors [127,128,129,130]. The rates of post-operative bacterial meningitis for patients undergoing EEA are reported to be 0% to 0.69%. Reported antibiotic protocols varied and included cefazolin or ampicillin/sulbactam; for patients with sensitivities to first-line agents, antibiotic protocols have included vancomycin, clindamycin, or clarithromycin. One study’s regimen was amikacin with either ceftazidime or ceftriaxone [127]. Antibiotic regimens ranged from a single dose 30 min prior to surgery to a 3 day duration [127,128,129,130]. Existing literature does not support an association between choice of antibiotic agent, duration of antibiotic regimen, and rates of post-operative meningitis. Recently a randomized control trial studied 113 patients undergoing standard transsphenoidal surgery for pituitary tumors and found that postoperative prophylactic oral antibiotics did not result in better sinonasal quality of life relative to placebo [131].

## 9. Other Complications and Peri-Operative Considerations

### 9.1. Venous Thromboembolism

While rare in the population of patients undergoing EEA, venous thromboembolism (VTE) may occur especially in patients who are older, have coagulopathy disorders, or have peripheral vascular disease. In these patients, care should be given to place sequential compression devices and utilize chemical VTE prophylaxis when appropriate [132]. This is especially true in any patient who experiences another complication from surgery, such as CSF leak or CN dysfunction, requiring extended immobility and hospital length of stay.

### 9.2. Cerebral Infarction

Cerebral infarction may develop from vasospasm, subarachnoid hemorrhage, direct vessel injury, or changes in volume or electrolyte status. One institution’s practice for minimizing the risk of vasospasm is irrigation of the intracranial space with normal saline after tumor resection and hemostasis, followed by application of cottonoid pledgets soaked with a vasodilating agent [133]. The authors also utilize intravenous nimodipine in the post-operative period along with ample hydration in cases of presumed or confirmed vasospasm.

### 9.3. Pneumocephalus

Pneumocephalus may develop in both an acute and delayed fashion after EEA [134,135]. Development of pneumocephalus may be through a one-way valve in which air enters from the extracranial space through CSF drainage (occurring through mechanisms such as nose blowing or insufflation of air after dural closure). Alternatively, when CSF is removed (for example through lumbar drainage) air may enter and displace the CSF. Minimizing the risk and amount of pneumocephalus is through careful LD management and implementation of patient sinus precautions.

### 9.4. Peri-Operative Considerations

Other measures to improve peri-operative safety include the administration of nasal decongestants to reduce bleeding, use of image guidance to assist in intraoperative navigation, early preparation of potential tissue graft sites, proper placement of endotracheal and orogastric tubes to facilitate surgical access, and appropriate bed/patient orientation to facilitate surgeon ergonomics [136]. Consideration should be given to whether the nature of the planned surgery warrants placement of arterial lines or urinary catheters. For example, anticipated manipulation of the posterior pituitary gland would place the patient at risk of intra- or post-operative DI or SIADH, prompting strict fluid intake and output measurements during and after surgery [136].

## 10. Sinonasal Morbidities and Postoperative Quality of Life

In addition to prevention of major complications in skull base surgery, there is an increasing recognition of the morbidities associated with the EEA and their potential impact on patient quality of life. Potential sinonasal morbidities may result from direct surgical trauma to the sinonasal corridor or alterations of sinonasal anatomy intrinsic to a surgical approach. These morbidities may include decreased olfaction, sensory changes, nasal crusting, nasal obstruction, rhinosinusitis, mucocele formation, and cosmetic deformities [137,138,139,140,141]. 

A recent systemic review with meta-analysis demonstrated that patients undergoing EEA often experience a worsening of sinonasal quality-of-life scores as measured by the Sino-Nasal Outcome Test (SNOT-22) instrument one month postoperatively followed by statistically improved scores at six- and 12-months [142]. Patients with worse preoperative sinonasal qualify-of-life scores demonstrated significant SNOT-22 score improvements as early as 12-weeks postoperatively that were sustained in long-term follow-up. For patients undergoing EEA for pituitary adenoma, no long-term detriment in sinonasal quality of life was reported in a single-center series of 50 patients over an average follow-up period of 24 months [143]. In another study of 81 pituitary adenoma patients, a transient worsening of Anterior Skull Base Questionnaire (ASBQ) and SNOT-22 scores was observed three weeks postoperatively. Beginning 12 weeks postoperatively, SNOT-22 scores returned to preoperative baseline and ASBQ scores improved beyond their preoperative baseline [144]. In a multi-institutional study of 100 that underwent EEA for pituitary adenoma, sinonasal quality of life was found to nadir at two weeks followed by a recovery at approximately three months [145].

### 10.1. Nasoseptal Flap Reconstruction

Nasoseptal flap reconstruction (NSF) is a critical advancement that revolutionized endoscopic skull base surgery but is associated with its own morbidities such as nasal crusting, olfaction changes, mucocele formation, septal perforation, septal flap necrosis, and external nasal deformities [146]. Techniques to reduce NSF morbidity have included placement of a harvested free mucosal graft on the donor site which in one study reduced nasal crusting six weeks postoperatively from 85% to 5% [147]. For standard sellar defects, free mucosal graft reconstruction can successfully prevent postoperative CSF leaks while having a more favorable donor site morbidity profile relative to NSF reconstruction [148]. Nasal access guides have been developed to reduce septal mucosa trauma which by reducing trauma to the contralateral septal mucosa after NSF elevation may reduce the risk of septal perforation [149]. Mucocele formation is favored to be secondary to incomplete sinonasal mucosal removal at the NSF recipient bed [148].

### 10.2. Olfaction

Olfactory dysfunction after EEA is thought to correlate with the extent of disease burden and surgical involvement of the olfactory tracts [137,150,151,152,153]. Postoperative olfactory morbidity varies with operative technique and approach. With respect to middle turbinate resection, it does not appear to be associated with long-term olfactory dysfunction [148]. NSF elevation is not clearly associated with long-term olfactory loss. In a pooled meta-analysis of patients undergoing EEA for sellar and parasellar lesions there was no significant difference in preoperative and postoperative University of Pennsylvania Smell Identification Testing (UPSIT) results regardless of reconstruction technique [152]. A randomized controlled trial found no decrease in UPSIT scores relative to preoperative baseline scores for patients who received NSF elevation during EEA regardless of whether electrocautery or cold steel techniques were used [154]. Another study showed that patients who received a NSF for EEA reconstruction had worsened subjective olfaction one month postoperatively but had returned to preoperative baseline olfaction by three months [155]. In a series of 14 patients undergoing surgery for esthesioneuroblastoma, in which all patients are expected to be anosmic post-operatively, 43% of patients with one preserved olfactory bulb had residual smell function after EEA and 14% of patients had normal or only mildly reduced smell [156].

### 10.3. Sensory Changes

In a study of patients undergoing EEA for repair of lateral pterygoid recess encephaloceles, 75% of patients developed at least transient hypesthesia, 62.5% of patients developed paresthesias, and 50% of patients developed dry eye [157]. However, in a series of 37 patients undergoing an endoscopic transpterygoid approach for tumor resection, 70.3% of patients had sacrifice of the vidian nerve but 38.5% of these patients complained of dry eye postoperatively [158]. The vidian nerve and CN V2 must often be resected as part of a surgical approach but there are recent efforts to describe vidian and greater palatine nerve-sparing approaches or the use of a lateral trans-orbital approach in lieu of the endoscopic transpterygoid approach for lateral encephalocele repair [159,160].

### 10.4. Aesthetic

Most quality-of-life studies have emphasized sinonasal morbidity however there has been recent emphasis in the aesthetic complications of the EEA [161,162]. External nasal deformities such as saddle nose deformity are more common than previously thought and seem to be most associated with NSF reconstruction and non-pituitary neoplasm surgery. The exact etiology of nasal dorsum collapse is not known but proposed techniques for minimizing its risk include preservation of septal mucosa and general minimization of NSF comorbidities through techniques such as free mucosal grafts, “reverse flap” technique, xenoplastic grafting, and silastic splinting [147,161,163,164,165].

## 11. Conclusions

Despite tremendous advances in the scope and safety of endoscopic skull base surgery, there remains significant room for improvement in our rates and management of complications. There are myriad potential complications and associated measures for not only recognizing them but also reducing their associated risk. Even in the most experienced hands, complications may still arise. Multi-disciplinary operative teams comprised of many individuals with a variety of experience and skill may lead to opportunities for mistakes to arise as well. For this reason, checklists have become a critical aspect of modern surgical care including skull base surgery. Within neurosurgery, checklists have been shown to reduce unplanned readmissions from 25% to 10% (*p* = 0.02) and wound complications from 19% to 8% (*p* = 0.04) [166]. A checklist for transsphenoidal surgeries has been developed with consideration given to different phases of EEAs [136]. Checklists help confirm necessary equipment is present and functional while promoting basic safety and team communication.

Despite the advances afforded by the EEA, there remain circumstances in which either a microscopic, open, or combined approach may be more favorable. For example, while the EEA technique does provide a direct trajectory to ventral skull base lesions, lateral extension of a tumor over the orbital roofs could favor an open approach when attempting complete lesion resection [167,168]. Additionally, other considerations such as preservation of olfaction could favor an open approach over EEA. Regarding the microscopic technique, a recent meta-analysis comparing microscopic and endoscopic techniques showed that the EEA does not increase gross total resection rates for pituitary adenomas [169].

Future directions for improving our understanding of complications associated with the EEA include investigation of various tissue sealants and packing materials available for skull base reconstruction, further defining the role of lumbar drainage in EEAs, and continuing to better define relevant endoscopic surgical skull base anatomy. Decision-making in endoscopic skull base surgery would benefit from a greater understanding of quality-of-life metrics and changes associated with the unique sinonasal morbidities arising from skull base lesions and treatment via the EEA. Additionally, how we perform the EEA will be shaped by upcoming technological advances inclusive of intraoperative robotics, augmented and virtual reality, and ultrasound/iCT/iMRI-based navigation [170,171,172,173,174,175].

There are several limitations to our review. We have chosen to use this manuscript as an opportunity to highlight the potential complications associated with the EEA and their avoidance. However, the present review does not describe complication management, a topic with a breadth of discussion beyond the scope of our manuscript. Complication management has been discussed previously in other publications [107,176,177,178,179,180,181,182]. Additionally, the present discussion is limited to the endoscopic endonasal technique but there are several circumstances in which alternative approaches such as the open or microscopic technique may represent more favorable surgical approaches.

The EEA has modernized skull base surgery and provided surgeons with a means for treating an ever-expanding number of pathologies. As adoption of the EEA grows, so too does the need for preventing, recognizing, and managing associated complications. The present review provides an overview of the most common complications associated with the EEA and measures for preventing them. Further research is required into strategies for reconstruction, endoscopic endonasal anatomy, and quality of life as it applies to the EEA.

## Data Availability

Not applicable.
